# Advanced human mucosal tissue models are needed to improve preclinical testing of vaccines

**DOI:** 10.1371/journal.pbio.3001462

**Published:** 2021-11-12

**Authors:** David Komla Kessie, Thomas Rudel

**Affiliations:** Chair of Microbiology, Biocenter of the University of Würzburg, Würzburg, Germany

## Abstract

There is a need for better models to improve preclinical testing of vaccines. This Perspective article argues that advanced mucosal human tissue models could present a solution to this pressing problem in the future.

## Teaser

Many vaccine candidates with promising results in preclinical testing fail in human trials. New complex human tissue models have the potential to improve the predictability of vaccine safety and efficacy in human clinical trials.

Vaccine development involves highly complex and expensive processes that have been classified by regulatory bodies into 2 stages: preclinical (in vitro and in vivo animal testing) and clinical (human). Potential vaccine candidates can fail at either stage. Before being introduced into the market, potential vaccines must be assessed for toxicity, immunogenic response stimulated, efficacy, and public health impact. This phase of the vaccine development process is usually done with 2D in vitro cultures of human cell lines as well as animal models such as mice and small primates. Proper formulation of vaccine candidates further requires a better understanding of host–pathogen interactions. Although useful, data generated using animal models do not directly translate to the human system. Considerable physiological and immunological disparities exist between humans and animals while 2D human cell cultures lack the complexity of the in vivo tissue. For example, Toll-like receptor expression on innate immune cells such as the dendritic cells critically dictate the type of immune response [1]. Toll-like receptors are a class of proteins expressed by host cells that detect structurally conserved microbe molecules such as lipopolysaccharides. Due to some of these discrepancies, many therapeutic and vaccine candidates that show great potential at the preclinical phase proved to be rather disappointing during human trials. There is, therefore, the need for improved test systems that capture the complexity of the tissue environment and provide data that are easily translated to the clinics.

**Fig 1:**
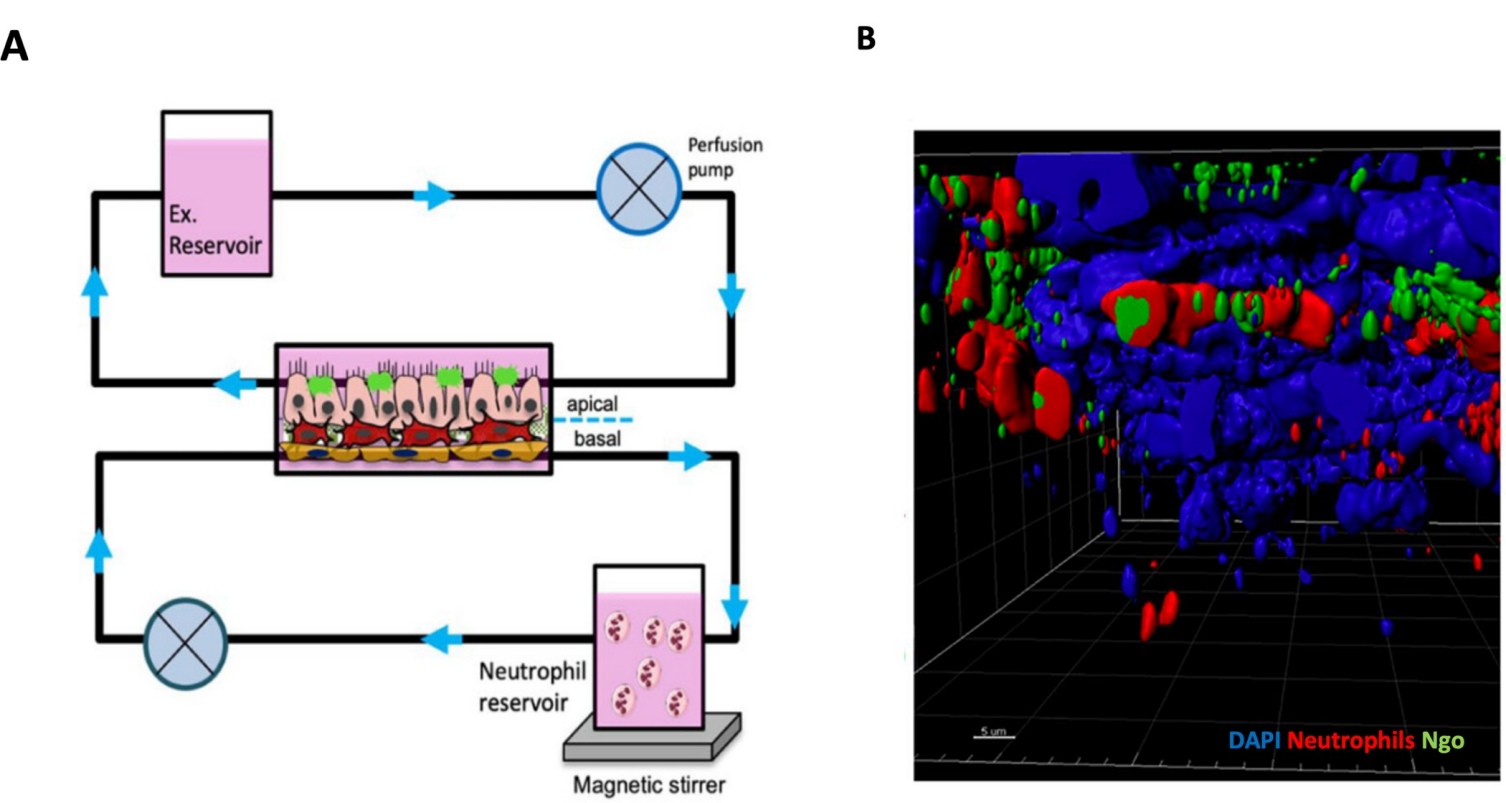
(A) Schematic of a perfusion-based bioreactor system that can be used for studying host–pathogen interaction as well as immune response to infection. This system is composed of a main chamber for holding the 3D human tissue model, an immune cell reservoir (neutrophils) and culture medium reservoir. (B) Reconstructed Z-stack of the confocal laser scanning microscopy images of an immunocompetent 3D human mucosa tissue model infected with *Neisseria gonorrhoeae* from the perfusion bioreactor system. (Adopted from Heydarian and colleagues [6]).

Current advances in tissue engineering and cell biology have provided many tools for the development of complex in vitro 3D test systems relevant for studying human diseases and vaccine development. These 3D models have high in vitro–in vivo correlation with the native tissues from which they are derived. They therefore have the advantage of assisting in better characterization and elucidation of the mechanism of host–pathogen interactions compared to 2D cultures of the same cells. Presently available 3D models include self-organizing and cell-supporting matrix models such as organoids and spheroids as well as scaffold based in vitro complex models [2]. The scaffolds usually consist of hydrophilic polymer chains that are cross-linked to form stable hydrogels to support cell growth. However, biological scaffolds obtained by decellularizing explanted organs such as small intestine of pigs, lungs, and liver also exist [3,4]. Further inroads have also been made with organ-on-chip technology to allow multiple organ systems to be connected on a flow cell, thus mimicking the connectivity between the various organ systems [5]. To make the in vitro models immunocompetent, methods to incorporate both humoral and/or cellular immune cells isolated from peripheral blood such as dendritic cells, neutrophils, macrophages, and T cells to static 3D cultures or via dynamic flow culture systems in perfusion bioreactors have been reported. The complexity of the models can and has been extended by coculturing epithelial cells, fibroblast, endothelial cells, and circulating immune cells in a perfusion bioreactor as shown in Fig 1 [6]. Perfusion systems also help to overcome the issue of hypoxia associated with 3D cultures. Significant strides in tissue engineering were made possible by advances in induced pluripotent stem cells, microfluidics, and the development of novel biomaterial scaffolds.

Infections usually start at exposed mucosal surfaces of the respiratory tract, gut, and reproductive tracts. Currently, in vitro 3D mucosal models have successfully been implemented to elucidate the subtle differences in the interaction of various pathogens and their virulence factors with their human host. The role of the mucosa in the establishment of immunity against infections such as SARS-CoV-2, influenza, or even strictly human-specific pathogens like measles virus, *Neisseria gonorrhoeae*, and *Streptococcus pneumoniae* and other pathogens can be assessed using these in vitro 3D human mucosal models. Potential applications of these 3D in vitro models during the vaccine development include assessment of toxicity testing and the type, quality, and quantity of the immune response elicited. These models can also be applied to study the cell-mediated immune response as well as immune interference and/or cross-reactions between coadministered or other vaccines. The data generated may directly correlate with the human response, thus speeding up the vaccine development process. There is, therefore, an increasing interest in the potential impact of these models in the vaccine development process. This interest is reflected in the addition of the advanced human tissue models work package to the recently installed Inno4Vac consortium under the EU-funded Innovative Medicine Initiative (IMI), which is aimed at accelerating vaccine development and manufacture [7].

Although these proposed ideas are provocative, we are still decades away from a fully integrated system similar to the human body due to currently existing challenges related to integration and long-term culture of the models with immune cells. Furthermore, a connected multi-organ system consisting of the plethora of human organs/tissues is currently not possible due especially to differences in cell culture media formulations for the different tissues. The development of a universal cell culture media formulation like the human blood capable of sustaining long-term culture, proliferation, and differentiation of all the different cell types will be critical to attain this goal. However, the currently available models can be used to assess sera from vaccinated individuals as well as convalescent patients for their protective effect against reinfections. They can also be utilized for toxicity tests, assess viral vector infectivity, and lipid nanoparticle transfection efficiency. Coupled with in silico predictive systems, in vitro 3D human tissue models can provide a platform for high-throughput screening of potential therapeutics and vaccine candidates. There is, therefore, optimism that complex immunocompetent 3D in vitro models will provide better models for early identification of toxicity and complications in the vaccine and drug development process, thus saving cost and time.

Finally, for these in vitro 3D models to be fully integrated into the vaccine development process, advancement of the models by incorporating multiple cellular components, improving microfluidic systems alongside other state-of-the-art technologies to overcome the current shortcomings are needed to improve their predictability and reliability.
